# Tracking single-molecule ferritin reassembly and disassembly using polymer-coated nanopores

**DOI:** 10.1039/d5nr02885j

**Published:** 2025-12-01

**Authors:** Mahya Assadipapari, Alireza Soleimanian, Max Adam, Jiali Li, Arman Yousefi, Saaman Zargarbashi, Lei Xu, Max F. K. Wills, Hesna Kara, Marina Santana Vega, Alasdair W. Clark, Andrew J. Hudson, Jian-An Huang, Mohsen Rahmani, Cuifeng Ying

**Affiliations:** a Advanced Optics and Photonics Laboratory, Department of Engineering, School of Science and Technology, Nottingham Trent University Nottingham UK cuifeng.ying@ntu.ac.uk; b Research Unit of Health Sciences and Technology (HST), Faculty of Medicine, University of Oulu Oulu Finland; c Department of Physics, University of Arkansas Fayetteville AR USA; d Department of Chemistry, Physical and Theoretical Chemistry Laboratory, University of Oxford Oxford UK; e Kavli Institute for Nanoscience Discovery, University of Oxford Oxford UK; f School of Physics and Astronomy, University of Nottingham Nottingham UK; g Leicester Institute of Structural and Chemical Biology Henry Wellcome Building University of Leicester Leicester UK; h Department of Molecular and Cellular Biology Henry Wellcome Building University of Leicester Leicester UK; i Biomedical Engineering Research Division, James Watt School of Engineering, University of Glasgow Glasgow UK; j School of Chemistry, University of Leicester Leicester UK; k Research Unit of Disease Networks, Faculty of Biochemistry and Molecular Medicine, University of Oulu Oulu Finland; l Biocenter Oulu, University of Oulu Oulu Finland

## Abstract

Ferritin is a multimeric protein that stores and releases iron, emerging as a promising candidate for nanomedicine, particularly in targeted drug delivery. Its ability to disassemble and reassemble under specific environments is crucial for encapsulating and releasing therapeutic payloads. In this work, we monitor ferritin reassembly and disassembly in real time at the single-molecule level using polymer-coated solid-state nanopores. The coating enabled continuous ion current recording of ferritin fragment translocations for up to 1 hour without clogging, overcoming a major challenge of uncoated nanopores. This long-term recording allowed us to track the full reassembly and disassembly processes. We show that while traditional population-based analysis can identify the presence of fragment mixtures during both reassembly and disassembly processes, it is unable to discriminate individual subunits due to heterogeneity in ferritin fragment mixtures. In contrast, using individual event analysis, we determined the volume and shape of individual fragments using single translocation events, enabling the identification of intermediate subunits (*e.g.*, 4-, 6-, 8-, 10-,12-, and 16-mers) during the reassembly and disassembly processes. Through real-time tracking of ferritin reassembly and disassembly dynamics, this approach – combining nanopore coating and individual event analysis – demonstrates potential to characterize single proteins within a mixture, determine their compositions, and track reaction processes in real time.

## Abstract 2

This study uses polymer-coated SiN_*x*_ nanopores and individual-event data analysis to track real time volume and shape changes of ferritin fragments during reassembly and disassembly, offering a powerful method for probing dynamic protein processes.

## Graphical abstract

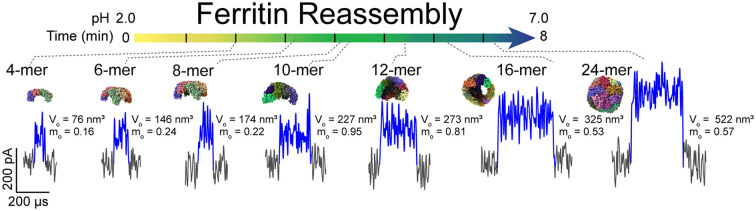

## 1. Introduction

Ferritin is a spherical protein composed of 24 subunits, forming a hollow nanocage with an outer diameter of 12 nm and an inner cavity of 7–8 nm.^1^ Its stability up to 85 °C, along with its capacity for reversible disassembly and reassembly, positions ferritin as an ideal candidate for use as a nanocarrier. These properties enable it to encapsulate various therapeutic agents, including anticancer drugs and imaging probes.^2,3^ A comprehensive understanding of ferritin's structural changes in response to environmental conditions and its assembly/disassembly processes is essential for advancing its applications in medicine and drug delivery.

Various ensemble techniques, such as velocity measurements, circular dichroism,^4^ synchrotron small-angle X-ray scattering,^5^ and time-resolved methods,^6^ have elucidated ferritin's disassembly and reassembly pathways by identifying structural intermediates. However, ensemble averaging limits its ability to capture disassembly and reassembly dynamics.^7,8^ Therefore, single-molecule approaches are essential to reveal how ferritin responds to different environments, enhancing its efficacy as a nanocarrier for drugs and bioactive molecules.^9^ Techniques like *in situ* AFM, bimodal magnetic force microscopy,^10^ high-speed AFM,^11^ MD simulations,^12^ graphene-liquid-cell STEM,^13^ and FRET^14^ have explored ferritin dis-/reassembly. Yet, these methods are invasive (*e.g.*, AFM), limited in duration (MD, ∼200 ns (ref. 12)), or require site-specific labelling (FRET). Our group previously demonstrated label-free, real-time monitoring of the conformational dynamics of ferritin during the iron loading process using plasmonic nanotweezers.^15^ Building on this work, we further revealed the stepwise-cooperative disassembly of ferritin under acidic conditions with single-molecule resolution.^16^ Although plasmonic nanotweezers have provided insight into the dynamics and pathway of ferritin disassembly, they suffer the limitation of inherent low throughput.^17^ The use of solid-state or synthetic nanopores has emerged as a promising label-free, single-molecule technique that enables the analysis of ultra-low sample volumes and concentrations.^18,19^ Using solid-state nanopores, Yin *et al.* discriminated ferritin and apo-ferritin based on their interior structural differences.^20^ They further engineered ferritin-based transmembrane nanopores as a single-molecule sensor for amino acid and peptide detection.^21^

The nanopore setup consists of a nanometer-scale pore within a membrane that separates two compartments filled with electrolytes. When a voltage is applied across the membrane, ions migrate through the nanopore, generating an ionic current.^22^ As a nanoparticle passes through the nanopore, it alters the pore conductivity, resulting in a change in the ionic current.^23,24^ Each current change, referred to as a resistive pulse, is characterized by the current blockade amplitude (Δ*I*) and dwell time (*t*_d_).^25–27^ These parameters provide information about the particle's shape, size, and charge.^28^ High-precision nanopore fabrication methods, including ion-beam,^29^ electron-beam,^30^ controlled breakdown techniques,^24,31,32^ reactive ion etching^33–35^ and laser thinning/laser-assisted breakdown,^36–39^ have enabled precise control over nanopore size to allow characterization of biomolecules across a wide range of sizes, such as DNA,^23,40–43^ RNA^40,41^ and proteins.^25,44–47^ Nanopore sensing typically relies on population-based analysis, assuming a single analyte type and characterizing it statistically across many translocation events. This method averages out heterogeneity, especially in mixtures of similarly sized analytes. In principle, each resistive pulse carries shape and size information for the translocating analyte.^28^ However, accurate extraction requires long dwell times (*e.g.*, *t*_d_ > 100 µs) to gather enough data points for reliable shape estimation.^25^ Acquiring a sufficient number of such long events in nanopore experiments is often challenging due to pore clogging caused by nonspecific interactions between the pore sidewalls and biomolecules.^26,48,49^ This clogging restricts the application of solid-state nanopores for monitoring long-term biological processes like protein disassembly, reassembly, aggregation and fibrillation. Surface functionalization of the nanopores, such as atomic layer deposition,^50,51^ surfactant-based surface modification,^52^ salinisation,^53^ and chemical deposition of various polymers,^42^ has effectively reduced these interactions, allowing extended interrogation of various analytes.^54,55^

While individual event analysis using coated nanopores has revealed the heterogeneity of the protein mixture,^56^ the potential for time-dependent structural characterization during reactions remains unexplored. Here, we have extended this approach to dynamically monitor and quantify individual fragments across the full time course of ferritin reassembly and disassembly. We have revealed that the reassembly process sequentially yields the predominant intermediates, 6-, 8-, 12- and 16-mers, within ∼8 minutes. In contrast, ferritin rapidly disassembles (<1 minute) into intermediates such as 16- 12-, 10-, 8-, 6- and 4-mers in a high-salt and harsh acidic environment. Tetramer and dimer interactions appear to play a key role in the formation of larger subunits during this process. The approach presented here provides a robust framework for tracking the composition within protein mixtures and offers detailed insights into the disassembly and reassembly processes of ferritin proteins at the single-molecule level.

## 2. Experimental section

### Chemicals

PBS tablets (ref. P4417), glycine (ref. G7126), horse spleen ferritin (referred to as ferritin in this work, ref. F4503), KCl (ref. 9541), and H_2_O_2_ solution (ref. 31642) were purchased from Sigma-Aldrich. Poly-l-lysine-grafted-PEG (PLL-*g*-PEG, *x* = 20, MW = 2000, % PEG = 10) was purchased from Nanosoft Polymers.

### Nanopores

SiN_*x*_ nanopores were fabricated by ion beam sculpting as described previously.^29^ Briefly, we first drilled a ∼100 nm initial pore in a 250 nm thick, free-standing SiN_*x*_ membrane supported on a 3 mm × 3 mm square silicon chip. Subsequently, the pores were refined to target diameters (*d*_p_) of 20–40 nm and effective lengths (*l*_p_) of 20–30 nm using feedback-controlled Ar^+^ ion beam sculpting. Fig. S1 shows the TEM images of nanopores used in this study. For reassembly experiments, a nanopore with a diameter of 21 nm and a length of 30 nm was employed. For the disassembly experiments, a nanopore with a diameter of 24 nm and a length of 20 nm was used for dataset 1, while a nanopore with a diameter of 30 nm and a length of 30 nm was used for datasets 2 and 3. Additional details on nanopore geometry and fabrication are provided in Rollings *et al.*^57^

Before use, nanopore chips were cleaned in a hot (60–80 °C) Piranha solution (H_2_SO_4_ : H_2_O_2_, 3 : 1) for 40 minutes, followed by thorough rinsing with deionized water and drying with compressed air. The cleaned chip was placed between two PDMS layers with 1.2 mm holes, as shown in Fig. S2, with *cis* and *trans* reservoirs filled with experiment-specific electrolytes. All electrolytes were filtered through a membrane with 0.22 μm pore size before experimental use.

### Data recording

A dPatch® digital patch clamp amplifier (Sutter Instrument Co.) functions as both an amplifier and a digitizer to apply voltage and record current. The voltage was applied *via* two Ag/AgCl electrodes that were immersed in the reservoirs separately (Fig. S2). All data were recorded at a sampling rate of 500 kHz, with a Bassel filter at a cutoff frequency of 50 kHz.

### Surface coating

*In situ* surface functionalization was performed as described by Salehirozveh *et al.*^58^ A 0.01 mg mL^−1^ poly(l-lysine)-grafted-poly (ethylene glycol) (PLL-*g*-PEG) solution was prepared in 10 mM PBS (pH 7.4) and introduced into both compartments of the flow cell. Upon applying a bias of −750 mV, the current baseline reveals a sharp drop within one minute, indicating successful coating. The representative current traces of the coating process and coating stability under acidic conditions (pH 2.0) are shown in Fig. S3a and S3b, respectively. The coating thickness was calculated based on the current change before and after coating, assuming a purely geometric effect. Only when the estimated thickness fell within the range of theoretical polymer length (3 ± 1 nm) did we consider the pore to be fully coated (SI S4).

### Fully assembled ferritin

For experiments involving fully assembled ferritin within an uncoated pore, we filled *cis* (70 μL) and *trans* (800 μL) reservoirs with 1 M KCl, 10 mM PBS, adjusted to pH 5.8. Then the IV curve and current baseline were recorded at +100 mV. Subsequently, 4.1 μL of stock ferritin solution (145 μM) was added to the *cis* reservoir, resulting in a final concentration of 8 μM.

For experiments using a coated nanopore, the same experimental conditions were applied, except that the buffer pH was adjusted to 7.0 to allow subsequent disassembly process experiments.

### Ferritin reassembly

For reassembly experiments, we first recorded the IV curve and current baseline at +100 mV with the *cis* reservoir filled with 90 µL and the *trans* reservoir filled with 800 µL of 1 M KCl, 10 mM PBS at pH 7.4. We then prepared 10 μL of 40 μM ferritin solution by mixing 2.7 μL of stock ferritin (145 μM) with 7.3 μL of 1 M KCl PBS (pH 2.0). While continuously recording the current at +100 mV, we added 10 µL of the above solution to the *cis* reservoir, achieving a final ferritin concentration of 4 µM. We confirmed that mixing pH 2.0 and pH 7.4 buffers at a 1 : 5 ratio resulted in an electrolyte equilibrated to pH 7.0.

### Ferritin disassembly

We filled the *cis* (70 µL) and *trans* (800 µL) reservoirs with 1 M KCl, 100 mM glycine–HCl buffer at pH 2.0 and recorded the current baseline under −100 mV bias before adding protein solution. For experiment set 1, we mixed 2.5 µL of stock ferritin (initial concentration = 145 µM) into 70 µL of the electrolyte on the *cis* side, yielding a final concentration of 5 µM. All the currents were recorded under −100 mV bias. For experiment sets 2 and 3, we added 0.5 µL of stock ferritin directly instead, resulting in a final ferritin concentration of 1 µM while keeping all other conditions unchanged.

### Data analysis

All events were detected using the Translyzer package developed by Plesa *et al.*^48^ with a low pass filter of 15 kHz. For population-based analysis, we identified events meeting two criteria: (1) the current amplitude exceeds 5 times the baseline noise standard deviation (5 × *σ*), and (2) the dwell time is between 25 µs and 1000 µs. The 25 μs lower bound ensures sufficient temporal resolution for a system bandwidth of 50 kHz, while the 1000 μs upper limit excludes events arising from non-specific protein–nanopore wall interactions. We used the maximum current blockade value Δ*I*_max_/*I*_0_ of each event to calculate the probability density function (PDF) in Fig. 3 and 4. For individual event analysis, we set the event detection criterion to exceeding 6 × *σ* and the dwell time to between 100 µs and 1000 µs. This 100 µs lower bound ensures sufficient time for protein rotation within the pore. To estimate the shape and volume from individual events, we generated a PDF of the raw recorded current trace (filtered at 50 kHz) for every extracted event and calculated the empirical cumulative distribution function (CDF). We then fitted the CDF with the convolution model developed by Yusko *et al.*^26^ Only events fitted to CDF with an *R*-square (*R*^2^) > 0.99 and a Kolmogorov–Smirnov test *p*-value > 0.05 were considered for volume and shape estimation. These strict criteria result in relatively low event counts for individual event analysis compared to population-based analysis. Further details about individual event analysis are provided in SI S6.

### Mass photometry

We used Refeyn OneMP to record mass photometry data. The ferritin solution was prepared in 100 mM glycine–HCl at a concentration of 25 nM. For each measurement, 10 µL of this solution was added dropwise on a cover slide coated with a monolayer of perfluoroalkane, which enhances the performance of interferometric scattering microscopy (iSCAT) by surface passivation.^59^ Data were acquired over one hour, with measurements taken at 10 minute intervals. During each interval, 60 seconds of interferometric scattering data were recorded and used to generate a mass distribution using AcquireMP software from Refeyn Ltd.

## 3. Results and discussion

### Polymer-coated nanopore enables long-term translocation recording

We investigated the translocation of fully assembled ferritin through uncoated and PLL-*g*-PEG-coated SiN_*x*_ nanopores to assess the impact of surface functionalization on the nanopore translocation performance. We implemented *in situ* PLL-*g*-PEG coating on a fresh cleaned nanopore, following the method reported by Salehirozveh *et al.*^58^ (SI S3). PLL-*g*-PEG polymers electrostatically bind to the surface, while their PEG side chains create a highly hydrated interface that screens surface charges and form a hydrophilic, charge-neutral layer.^60^ This coating effectively minimizes protein adhesion and clogging, and by disrupting the formation of the electrical double layer, it further minimizes electroosmotic flow. To evaluate whether pores are successfully coated, we estimated the polymer layer thickness from the change in ionic current before and after coating, considering only geometric effects.^38^ The estimated thicknesses were then compared with the theoretical polymer length of 3.0 nm (Table S1). Pores were considered successfully coated when the calculated polymer length deviated by less than 25% of the theoretical value.

**Fig. 1:**
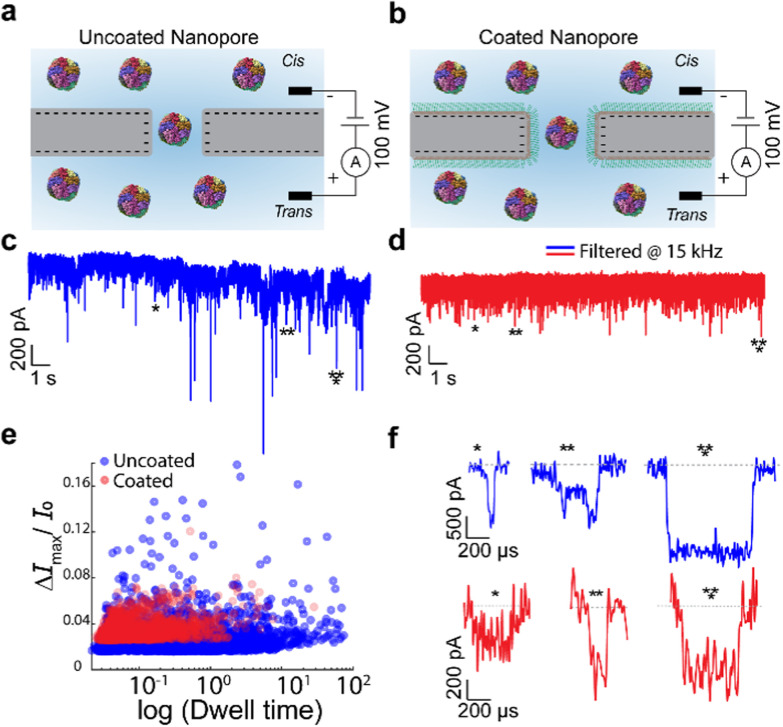
Ferritin translocation through a SiN_*x*_ nanopore. (a and b) Schematic of ferritin translocation (PDB: 1BFR) through an uncoated pore (a) and PLL-*g*-PEG-coated pore (b). (c and d) Representative 20 s current traces of ferritin translocation in the uncoated nanopore (c) and in the coated nanopore (d). Traces are digitally filtered at 15 kHz. (e) Scatter plot of dwell time *vs.* Δ*I*_max_/*I*_0_ for ferritin through the uncoated (blue circles) and coated (red circles) nanopores. (f) Zoomed-in profile for individual translocation events from panels c and d, digitally filtered at 50 kHz.

Fig. 1 shows a comparison of ferritin translocation through uncoated and coated nanopores. The translocation through uncoated and coated pores was conducted at pH 5.8 and pH 7.0, respectively. In the absence of the coating, the baseline current drifts over time and the blockade amplitudes vary widely (Fig. 1c). In contrast, the coated pore exhibits a stable baseline and consistent blockade amplitudes (Fig. 1d). This is further demonstrated by the event scatter plot (Fig. 1e), where compared to the coated pore, the uncoated pore produced a broader, more dispersed distribution, including a higher proportion of long-duration events (*i.e.*, >1 ms), arising from transient protein adhesion. Representative individual translocation events (Fig. 1f) for the uncoated pore show significant variations in both dwell time and current blockade amplitude, suggesting that free translocation is hindered by nonspecific interactions with the pore surface. In contrast, the coated pore exhibits more uniform and consistent current blockades, indicative of reduced surface adhesion and smoother translocation dynamics. This reduction in surface interactions by coating enables more reliable and consistent event statistics and enhances sensing accuracy in using nanopore technology.

**Fig. 2:**
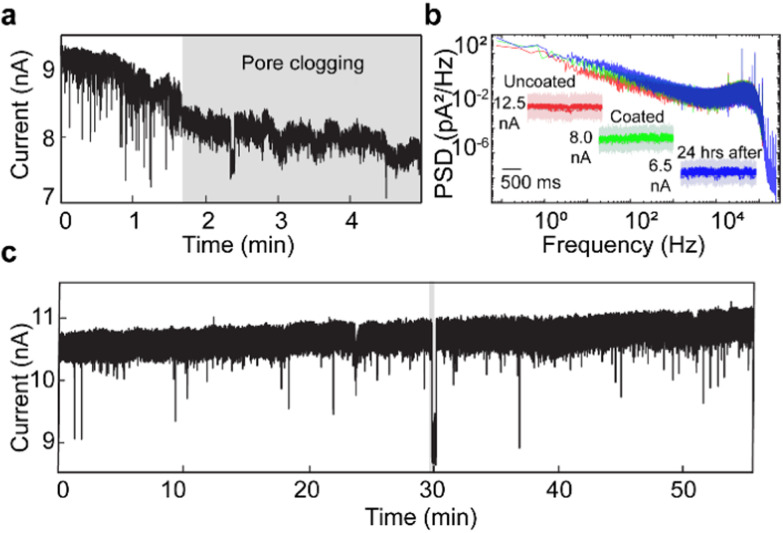
Protein translocation through nanopores before and after polymer coating. (a) Current trace of ferritin translocation through an uncoated nanopore at pH 5.8 (15 kHz).(b) Power spectral density (PSD) of 20 second baseline recordings before (red), immediately after (green), and 24 hours after (blue) PLL-*g*-PEG coating. Inset: raw and 15 kHz-filtered current baselines. (c) Current trace of ferritin disassembly through a polymer-coated nanopore during a gradual pH change from pH 2.0 to pH 7.0 over ∼1 hour (15 kHz).

To monitor reaction dynamics at the single-molecule level, achieving long-term translocation without clogging is essential. Fig. 2 demonstrates the capability of PLL-*g*-PEG coated nanopores for extended translocation experiments. In an uncoated nanopore (Fig. 2a), the baseline current dropped by ∼20% after 2 minutes, accompanied by a reduction in event frequency, suggesting pore clogging from protein adhesion.^54,61^ While reversing the voltage bias temporarily restored the baseline current, the clogging persisted after a minute (Fig. S4). In contrast, the functionalized pore exhibited a stable baseline for approximately one hour with an overall drift of only ∼5% (Fig. 2c), allowing continuous protein translocations. To assess the durability of surface functionalization, we compared the baseline current and the power spectral density (PSD) acquired before, immediately after coating and 24 h later (Fig. 2b). The coated nanopore exhibits slightly higher 1/*f* noise (<1 kHz) compared to the uncoated pore, likely due to flexible PEG side chains in solution and the formation of nanobubbles within the pore.^55,58,62^ However, the difference is negligible above 1 kHz, confirming minimal impact of high-frequency noise for protein characterization.^63^ We also observed an ∼19% decrease in the baseline for a coated pore after 24 hours of storage in water, which is attributed to polymer brush swelling in water.^64,65^ To ensure consistent experimental conditions, we conducted all experiments with freshly coated nanopores.

### Real-time monitoring of ferritin reassembly

**Fig. 3:**
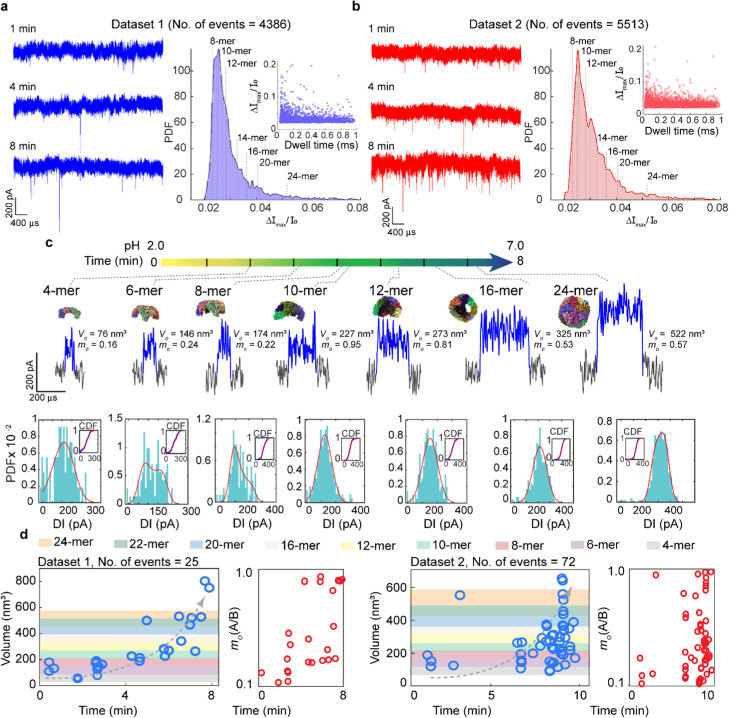
Ferritin reassembly monitored using PLL-*g*-PEG coated nanopores. (a and b) Representative 5 s current traces (15 kHz filtered) at the start, midpoint, and end of the 8 min reassembly process for dataset 1 (a) and dataset 2 (b), with corresponding histograms and probability density functions (PDFs) of Δ*I*_max_/*I*_0_. Dashed lines indicate the expected blockade amplitudes for different ferritin subunits, assuming a spherical geometry. Inset: scatter plot of dwell time *versus* Δ*I*_max_/*I*_0_. (c) Individual-event analysis for representative translocation events (50 kHz filtered) from dataset 1, corresponding to 4-, 6-, 8-, 10-, 12-, 16-, and 24-mers, along with PDFs and CDFs of their current blockade fitted using the convolution model (red curves). The fitted volume and shape are indicated for each event. (d) Estimated volume and shape (oblate, *m* < 1) for ferritin subunits over time.

To demonstrate the capability of monitoring dynamic reaction processes, we applied polymer-coated nanopores to track the ferritin reassembly process by recording translocation events immediately after adding disassembled ferritin (pH 2.0) to a pH 7.0 solution. Fig. 3a and b show representative 5 s current traces collected at 1, 4 and 8 minutes during an 8 minute recording for two datasets, together with their population-based analyses shown on the right. The current traces reveal a progressive increase in blockage current amplitude over time, reflecting reassembly progression. The broad distribution of Δ*I*_max_/*I*_0_ indicates a heterogeneous population of sizes corresponding to different subunits (*e.g.*, 8-, 10-, 12-, 14-, 16-, 20- and 24-mers) throughout the 8 minute recording. The sizes were estimated assuming only spherical geometry, as routinely done in population-based analysis. This assumption, however, introduces errors into volume estimation, as these intermediates adopt ellipsoidal geometries (see the structures analysed using ChimeraX in Table 1). To obtain accurate size estimations, we performed individual event analysis using the deconvolution model^25,26^ (see the details in SI S6) to determine the shape and volume of translocating species. Fig. 3c presents representative translocation events at different time points during the ferritin reassembly process, together with their corresponding oblate volume (and shape), PDF, and cumulative distribution function (CDF) derived from the current blockades. The estimated shape and volume, obtained from fitting the CDF to the deconvolution model, reveal a gradual increase in subunit size over time that reflects the progressive assembly of smaller fragments into larger subunits. We note that fittings were performed under the assumption of an oblate geometry (*m* < 1), based on structural information obtained from ChimeraX (Table 1). Fig. 3d plots the volume (and shape) as a function of time, with each circle representing the estimated volume (and oblate shape) from an individual translocation event. The significantly lower number of analyzed individual events compared with population-based analysis arises from the strict selection criteria applied, as detailed in the Experimental section. In addition to capturing structural heterogeneity, this approach enables continuous tracking of the evolving composition of ferritin fragments throughout the reassembly process.

**Table 1:** Extracted dimensions of ferritin subunits (PDB: 1BFR) using ChimeraX

Subunit	*M*W a (kDa)	Volume (nm^3^)	Length *A* (nm)	Diameter *B* (nm)	*m* (*A*/*B*)
Tetramer	76–84	83	2.5	9.4	0.27
Hexamer	114–126	125	3.6	11.1	0.32
8-mer	152–168	167	5.3	11.3	0.47
10-mer	190–210	209	5.3	12.1	0.44
12-mer	228–252	251	5.3	12.1	0.44
16-mer	304–336	330	6.9	8.0	0.86
24-mer	456–504	502	12.7	12.7	1.00

a Molecular weight (*M*W) was calculated based on the light-chain to heavy-chain of ferritin subunits.

We identified different ferritin fragments during the process, as indicated by the colour bands in Fig. 3d, which correspond to different ferritin subunits. In both datasets, we observed a gradual increase in subunit size, progressing toward the formation of a fully assembled ferritin nanocage (24-mer) over the 8 min recording period. It is noted that dimers were not detected as their current blockade (∼95 pA) is below the detection threshold of 156 pA (6 × 26 pA).

### Monitoring the ferritin disassembly process

**Fig. 4:**
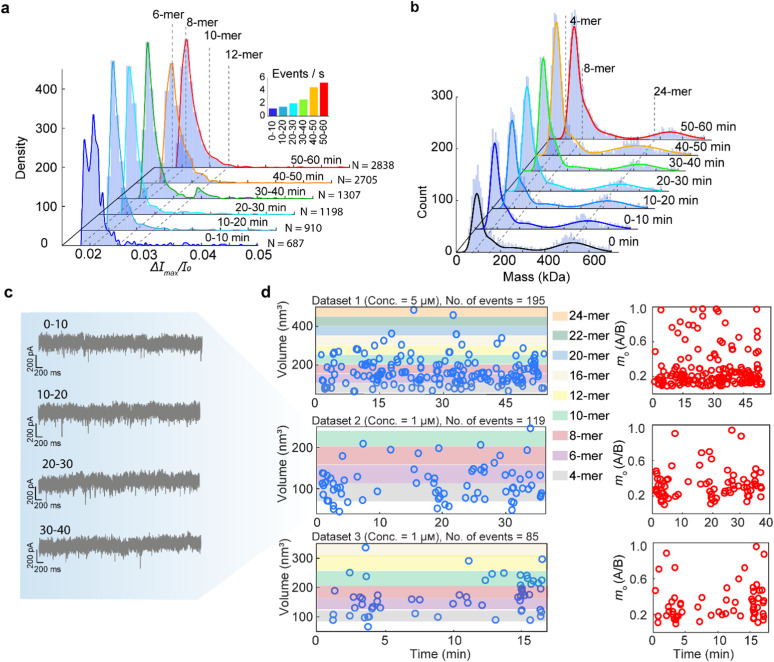
Ferritin disassembly monitored using PLL-*g*-PEG coated nanopores. (a) PDFs showing the distribution of Δ*I*_max_/*I*_0_ in 10 min intervals during disassembly. The PDFs were generated through population-based analysis of 9645 ferritin translocation events. The dashed lines indicate the peaks across all the PDFs. Inset: event rate for each 10 min interval. (b) PDFs of the molecular mass distribution acquired by mass photometry. (c) Representative 5 s current traces (15 kHz filtered) from dataset 2, recorded during the disassembly process at 0–10, 10–20, 20–30, and 30–40 minutes. (d) Estimated ferritin subunits’ volume and inferred shape (oblate, *m* < 1) over time.

We further apply polymer-coated nanopores for monitoring the reverse process—ferritin disassembly, by recording the ionic current under a −100 mV bias immediately after introducing ferritin into an acidic environment (pH 2.0). Similar to the procedure used for ferritin reassembly, a population-based analysis was performed on 15 kHz-filtered data to estimate volume changes over time. Fig. 4a presents the PDF of Δ*I*_max_/*I*_0_ for all events detected within each 10 minute interval during disassembly (dataset 1 of Fig. 4d, with the overall trace shown in Fig. 2c). The increasing event rate over time indicates the continuous breakdown of the intact nanocage into smaller fragments. Within the first 10 minutes of acidic exposure, the ferritin nanocage rapidly disassembled into smaller subunits compared to the assembled ferritin. We identified 6-mers and 8-mers as the most predominant fragments across all time intervals, evidenced by the two highest peaks in all PDFs, with 10-mers and 12-mers appearing less abundantly. Notably, dimers are predicted to be the predominant subunits under a harsh acidic environment.^12^ However, the corresponding Δ*I*_max_/*I*_0_ values for dimers and tetramers are estimated to be 75 and 150 pA, respectively. These values are at or below the event detection threshold of 220 pA (5 × *σ*, with *σ* = 44 pA).

As mass photometry offers complementary single-molecule size distribution analysis of label-free proteins,^66^ we employ it as an independent validation of ferritin disassembly under acidic conditions (pH 2.0). Fig. 4b tracks the size distribution at 10 min intervals upon introduction of ferritin into an acidic environment, confirming the rapid disassembly of the ferritin cage into small subunits within the first 10 minutes. The peaks of PDFs in Fig. 4b reveal tetramers (76–84 kDa) as the dominant fragment (highest PDF peak), followed by octamers (152–168 kDa) as the second dominant subunit. The ratio of these fragments increased over time as ferritin was continuously exposed to the harsh acidic environment. This behavior differs from the population-based nanopore analysis results in Fig. 4a, where subunits larger than octamers appear at a later phase. We note that mass photometry experiments to examine the disassembly of ferritin were conducted at a low ferritin concentration (25 nM) in a low-salt solution (100 mM KCl) to facilitate the separation of landing events for effective imaging,^17,67,68^ whereas for nanopore recording, we used 5 µM ferritin in a 1 M KCl solution to enhance the signal-to-noise ratio.^24^ The large fragments (>12-mer) in the nanopore experiment likely result from the high salt concentration, which elevates ionic strength, stabilizes the intermediates, and promotes subunit refolding as described by Stefanini *et al.*^4^

Compared to population-based nanopore analysis, mass photometry can detect smaller subunits (down to tetramers). However, it identified only tetramers, 8-mers and 24-mers across all time intervals, whereas nanopore measurements captured intermediate subunits between 6-mers and 12-mers, providing a higher resolution for similarly sized proteins. Neither population-based nanopore analysis nor mass photometry was able to identify dimers as the primary subunit formed during the disassembly process.

To resolve smaller subunits, such as tetramers, and track the dynamics of ferritin disassembly, we performed individual event analysis for three independent experimental datasets.

Fig. 4d shows plots of fitted volumes and shapes of individual ferritin subunits over time, as they translocate through a PLL-*g*-PEG-coated nanopore at pH 2.0. Compared with population-based analysis, this individual event analysis of dataset 1 enabled identification of smaller subunits, such as 4- and 6-mers. Across all three datasets, small subunits such as 4-, 6-, 8-, 10- and 12-mers were predominantly detected, underscoring their roles as key intermediates in the disassembly pathway. In dataset 2, an increase in event frequency was observed, as illustrated by the 5 s current traces recorded during ferritin disassembly (Fig. 4c), confirming the progressive breakdown of fully assembled ferritin into smaller subunits. However, we did not observe the transition from fully assembled ferritin to disassembled subunits. We hypothesize that this process occurs within a very short time window, too rapid to be captured by either mass photometry or nanopore measurements.

## 4. Conclusions

In this study, we employed PLL-*g*-PEG functionalized SiN_*x*_ nanopores to track the long-term reassembly and disassembly of ferritin *in situ*. Using the individual event analysis approach, we estimated the volume and shape of each translocation event, enabling us to monitor changes in protein shape and volume dynamically during ferritin reassembly and disassembly processes. During the reassembly process, we identified 4-mers, 6-mers, 8-mers, 10-mers, 12-mers, and 16-mers as stable intermediates, with the fully assembled ferritin cage forming within approximately 8 minutes. In contrast, under high salt conditions (1 M KCl), the disassembly process at pH 2.0 yielded stable intermediate fragments (4-, 6-, 8-, 10-, 12-, and 16-mers) rather than the gradual breakdown from large fragments to dimers typically observed at low salt concentrations.^16^

We conclude that despite the heterogeneity observed in these processes, 6-mers, 8-mers, 10-mers,12-mers, and 16-mers emerge as key intermediates formed during both the reassembly and disassembly of ferritin, as supported by individual event analyses.

One limitation of this work is the detection constraint for dimer fragments, whose event counts were underestimated due to the amplitude of current blockades being close to or below the detection threshold. Nevertheless, this work demonstrates the effectiveness of our approach in identifying individual proteins in a mixture across a wide range of sizes and shapes, while enabling the monitoring of changes in size and shape over extended durations without pore clogging. We believe that this approach can be applied to track various biological processes involving protein mixtures, such as protein assembly, disassembly, binding, and identification in complex mixtures. These processes are otherwise challenging to study using uncoated nanopores, which suffer from limited observation durations due to pore clogging, or traditional population-based analysis, which averages out the heterogeneity for individual molecules.

## Author contributions

M. A.: conceptualization, methodology, data curation, formal analysis, visualization, and writing (original draft). A. S.: data curation. J. L.: methodology and fabrication of SiN_*x*_ nanopores. A. Y.: conceptualization and writing (review & editing). S. Z.: conceptualization, formal analysis, and writing (review & editing). L. X.: supervision and writing (review & editing). M. F. K. W. and H. K.: data curation (mass photometry). M. S. V. and A. W. C.: sample preparation (mass photometry). A. J. H.: resources and writing (review & editing). J. H.: writing (review & editing). M. R.: funding acquisition, resources, and supervision. C. Y.: conceptualization, methodology, visualization, supervision, writing (review & editing), and funding acquisition. All authors have approved the final version of the manuscript.

## Conflicts of interest

The authors declare no conflicts of interest.

## Supplementary Material

NR-018-D5NR02885J-s001

## Data Availability

The data that support the findings of this study are available from the corresponding author upon reasonable request. Supplementary information (SI) is available. See DOI: https://doi.org/10.1039/d5nr02885j.
