# Supplementary material for: Tracking single-molecule ferritin reassembly and disassembly using polymer-coated nanopores
Source: Nanoscale. 2025 Dec 1;18(2):1045–54. doi: 10.1039/d5nr02885j (PMC12706752; doi:10.1039/d5nr02885j)
Supplement: NR-018-D5NR02885J-s001 [file NR-018-D5NR02885J-s001.pdf]

# Supporting Information

Supporting Information

## Tracking Single-Molecule Ferritin Reassembly and Disassembly Using Polymer-Coated Nanopores

Mahya Assadipapari,[a] Alireza Soleimanian,[b] Max Adam,[a] Jiali Li,[c] Arman Yousefi,[a] Saaman Zargarbashi,[a] Lei Xu,[a] Max F.K. Wills,[de] Hesna Kara,[de] Marina Santana Vega,[f] Alasdair W. Clark,[f] Andrew J. Hudson,[dg] Jian-An Huang,[bhi] Mohsen Rahmani [a] and Cuifeng Ying [a*]

[a] Advanced Optics and Photonics Laboratory, Department of Engineering, School of Science and Technology, Nottingham Trent University, Nottingham, United Kingdom.

[b] Research Unit of Health Sciences and Technology (HST), Faculty of Medicine, University of Oulu, Oulu, Finland.

[c] Department of Physics, University of Arkansas, Fayetteville, AR, United States of America

[d] Leicester Institute for Structural and Chemical Biology, Henry Wellcome Building, University of Leicester, Leicester, United Kingdom.

[e] Department of Molecular and Cell Biology, Henry Wellcome Building, University of Leicester, Leicester, United Kingdom.

[f] Biomedical Engineering Research Division, James Watt School of Engineering, University of Glasgow, Glasgow, United Kingdom.

[g] School of Chemistry, University of Leicester, Leicester, United Kingdom.

[h] Research Unit of Disease Networks, Faculty of Biochemistry and Molecular Medicine, University of Oulu, Oulu, Finland.

[i] Biocenter Oulu, University of Oulu, Oulu, Finland.

[*]Corresponding author email: cuifeng.ying@ntu.ac.uk

Please do not adjust margins



## S1. TEM Micrograph of $SiN_x$ Nanopores Fabricated by Ion Beam Sculpting

Figure S1 shows TEM images of SiNx nanopores fabricated by the ion beam sculpting method. These pores have diameters on the order of several tens of nanometers. At this size scale, the influence of the pore surface on ionic conductivity and current is negligible, because the region in which pore shape significantly affects ionic concentration and mobility, and therefore the ionic current, is limited to within less than 5 Å from the surface.[1] The pores also exhibit ellipsoidal shapes with ratios of short-to-long radius greater than 0.5. Under these conditions, the deviation in ionic conductance from that of an ideal circular pore remains below 4.5% in 1 M KCl, indicating that the presence of asymmetry in pore geometry does not meaningfully affect the ionic current measurements in this work.[2]

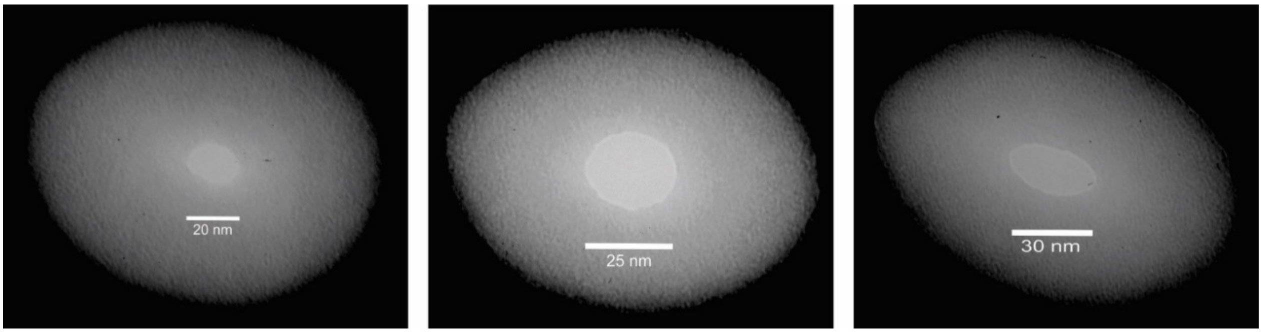


Figure S1: Transmission electron microscopy (TEM) images of three $SiN_x$ nanopores with diameters between 20 and 30 nm.

## S2. Nanopore Mounting in PDMS Flowcell

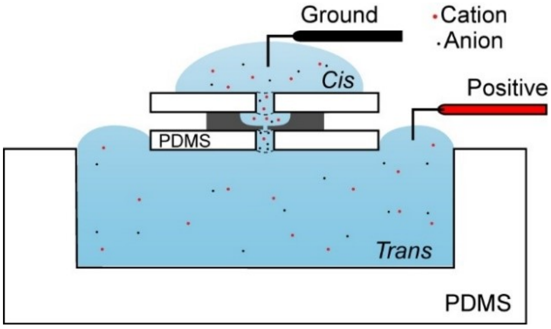


Figure S2: Schematic of a nanopore mounted in a PDMS flow cell with reservoirs filled electrolyte.



This journal is © The Royal Society of Chemistry 20xx

Please do not adjust margins

Please do not adjust margins



## S3. *In-situ* Surface Functionalization

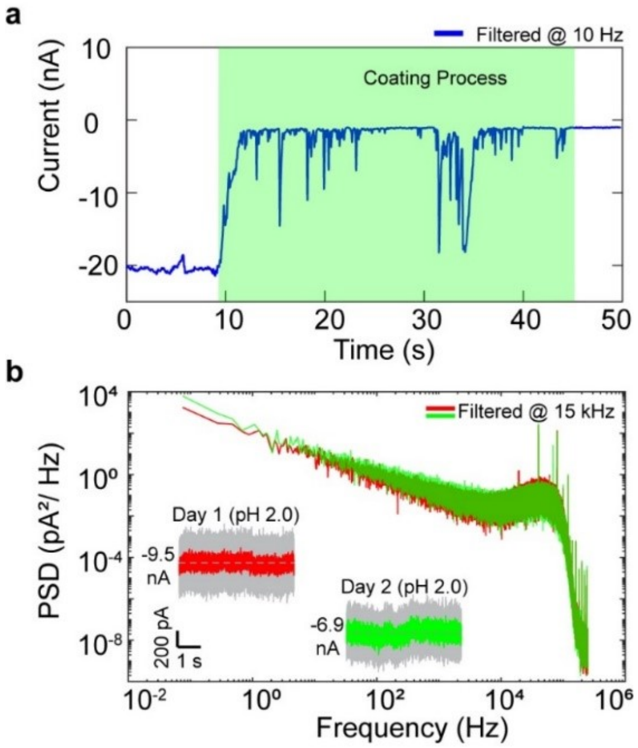


Figure S3: In-situ coating of $SiN_x$ nanopore. (a) Current trace of a nanopore in PLL-g-PEG solution under an applied voltage of -750 mV. (b) Power spectral density (PSD) of 20-second current baselines of polymer functionalized pore, recorded immediately after coating (red) and 24 hours after coating (green), both in 1M KCl at pH 2.0, with their corresponding 5-second current baselines in raw (gray) and filtered at 15 kHz (red and green) shown in the inset.

## S4. Pore Length and Polymer Thickness

We determined the radius of pore $r_p$ by area-equivalent defined as $r_p = \sqrt{r_{p1} r_{p2}}$, where $r_{p1}$ and $r_{p2}$ are two ellipsoid radii of the nanopore measured by TEM imaging.

We obtained the total resistance of the nanopore $R_{total} = {V}/{I_0}$, using the current baseline ($I_0$) and the applied voltage ($V$). The length of the nanopore $l_p$ was calculated by the Equation S1 as described in detail in ref.[3]

$$R_{total} = R_{pore} + R_{access} = \rho\left(\frac{l_p}{\pi(r_p^2)} + \frac{l_c}{\pi(r_c^2)}\right) + \rho(\frac{1}{2r_p} + \frac{1}{4r_c}) \quad \text{(S1)}$$

where $R_{pore}$ presents the resistance to ionic current through the pore itself, and $R_{access}$ denotes the resistance to ion movement at both the entrance and exit of the pore.

According to the experimental conditions:

$\rho$ = 0.092 (Ωm) as resistivity of 1M KCl solution.

$l_c = 275\ (nm) - l_P$ as channel length (275 nm is the $SiN_x$ membrane thickness).

$r_C = 50$ (nm) as channel area-equivalent radius.



Please do not adjust margins

Please do not adjust margins



After coating, we obtained the resistance of coated nanopore, $R_{total\_coated}$. We then added the polymer thickness to the geometry and $r_{p\_coated} = r_p - l_{poly}$ and $l_{p\_coated} = l_p + 2l_{poly}$ and solve the Equation S1 to obtain the $l_{poly}$. In Table S1, we have summarized the coating calculations for different pores. In this work, we only used fully coated pores with calculated polymer lengths deviating by less than 25% from the expected value (3.0 nm).

Table S 1: Coating length calculation for different pores using 1M KCl PBS (pH 5.7) under 100 mV electrical voltage

| No. | Rp (nm) | $I_{before}$ (nA) | $I_{after}$ (nA) | $l_p$ (nm) | $l_{poly}$ (nm) | Status |
|---|---|---|---|---|---|---|
| 1 | 15.0 | 13.5 | 8.0 | 33.0 | 3.3 | Fully Coated |
| 2 | 22.0 | 27.0 | 20.0 | 26.0 | 2.8 | Fully Coated |
| 3 | 13.2 | 7.6 | 8.0 | 30.0 | 0.0 | Not Coated |
| 4 | 14.2 | 10.0 | 7.5 | 33.0 | 2.8 | Fully Coated |
| 5 | 13 | 11.2 | 8.0 | 31.1 | 1.9 | Partially Coated |
| 6 | 20.0 | 22.0 | 16.0 | 30.0 | 2.8 | Fully Coated |
| 7 | 14.0 | 13.5 | 8.0 | 27.6 | 3.08 | Fully Coated |
| 8 | 14.2 | 11.3 | 10.6 | 32.0 | 0.4 | Partially Coated |
| 9 | 12.0 | 12.5 | 8.0 | 20.0 | 2.3 | Fully Coated |
| 10 | 14.0 | 17.0 | 11.0 | 31.0 | 3.1 | Fully Coated |
| 11 | 10.5 | 7.5 | 4.0 | 30 | 2.6 | Fully Coated |
| 12 | 11.0 | 7.9 | 3.5 | 30.5 | 3.5 | Fully Coated |
| 13 | 15.0 | 15.0 | 15.0 | 27.6 | 0.0 | Not Coated |

## S5. Pore Clogging Persists After Reverse the Voltage Bias

Figure S4 shows the ionic current trace measured at +100 mV during ferritin translocation through a bare pore. After two minutes, the voltage was reversed to -100 mV to sweep any clogged ferritin within the bare pore. The voltage was then returned to the original +100 mV, restoring the ionic current baseline.

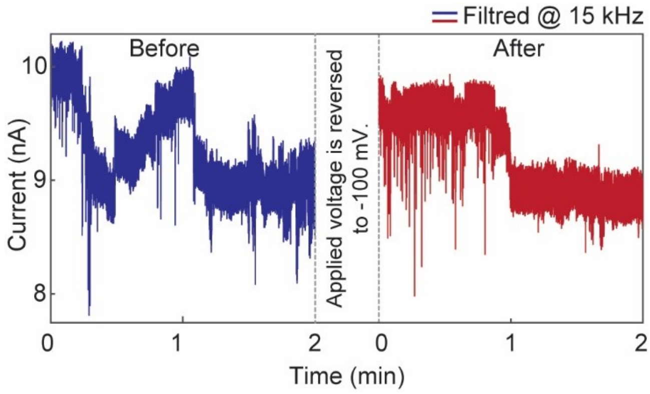


Figure S4: Filtered ionic current trace (15 kHz) recorded before and after reversing the applied bias voltage from +100 mV to -100 mV.

## S6. Estimation of Protein Shape and Volume

Resistive pulse nanopore sensing depends on ionic flux, which is linked to the presence of conductive, homogeneous electrolyte and non-conductive objects that pass through it. In the context of a uniform electric field and a cylindrical pore channel, each protein that is drawn into the pore by electrophoretic force alters the electric field. This distortion leads to a reduction in ionic current as the conductive electrolyte is displaced. Furthermore, ellipsoid particles can create fluctuations in the ionic current due to their varied orientations as depicted in Figure S5. This variation is characterized by a parameter known as the electrical shape factor ($\gamma$), which is influenced by the length-to-diameter value ($m$), as well as its orientation related to the electric field

 This journal is © The Royal Society of Chemistry 20xx

Please do not adjust margins

within the pore channel. Ref. [3,4] have provided a thorough and comprehensive explanation for proteins' shape and size estimation.

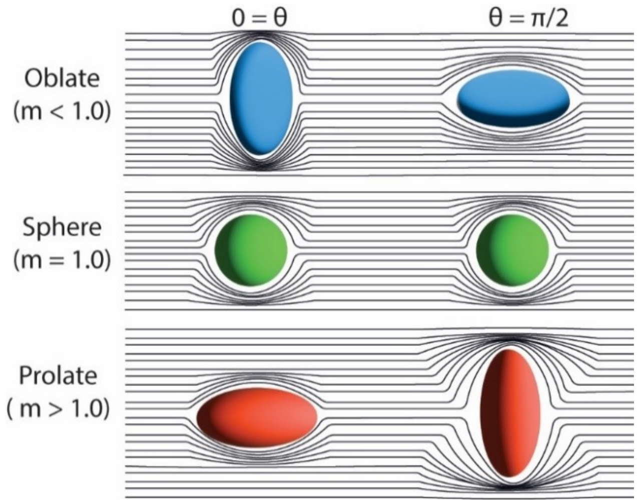


Figure S5: Electric field perturbation induced by oblate, spherical, and prolate ellipsoids in parallel and perpendicular orientations.

Here, we outline brief formulas that are used in our data analysis. Equation S2 represents the relation between magnitude of normalized current blockade ($\Delta I/I_0$) to the particle's volume ($\Lambda$) and electrical shape factor (γ). [3,4]

$$\frac{\Delta I}{I_0} = \frac{4\Lambda\gamma}{\pi d_p^2\left(l_p + \frac{\pi}{4}d_p\right)} \qquad \text{(S2)}$$

For spherical particle, $\gamma = 1.5$ and $\Delta I/I_0$ is proportional to the non-conductive particle's volume ($nm^3$). In this context, the minimum ($\Delta I_{min}$) and maximum ($\Delta I_{max}$) current blockades are associated with excluded volume of protein ($\Lambda$), and minimum and maximum electrical shape factor ($\gamma$) which is subsequently depends to $m$. The electrical shape factor is a function of angle between the axis of protein's shape symmetry and electrical field, as mentioned in Equation S3:

$$\gamma(\theta) = \gamma_{\perp} + (\gamma_{\parallel} - \gamma_{\perp})\cos^2(\theta) \qquad \text{(S3)}$$

$\vartheta$ is the angle between the axis of symmetry of the protein and the electric field. $\gamma_{\parallel}$ and $\gamma_{\perp}$ are defined for parallel and perpendicular orientations relative to the electric field, respectively. They are related to the depolarization factors ($\gamma_{\parallel} = \frac{1}{1-n_{\parallel}}$, $\gamma_{\perp} = \frac{1}{1-n_{\perp}}$), and subsequently to $m$. for prolate shapes ($m > 1$) we have:

$$n_{\parallel} = \frac{1}{m^2-1}\left(\frac{m}{\sqrt{m^2-1}} - \ln\left(m + \sqrt{m^2-1}\right) - 1\right) \qquad \text{(S4)}$$

And for oblates ($m < 1$):

$$n_{\parallel} = \frac{1}{1-m^2}\left(1 - \frac{m}{\sqrt{1-m^2}}\cos^{-1}(m)\right) \qquad \text{(S5)}$$

Then the perpendicular depolarisation factor is calculated by the Equation S6:

This journal is © The Royal Society of Chemistry 20xx

Please do not adjust margins



$$n_{\perp} = \frac{(1 - n_{11})}{2} \quad \text{(S6)}$$

For a prolate spheroid (*m* >1), the minimum and maximum of γ are:

$\gamma_{min} = \gamma_{\parallel} (when\ \theta = 0)$, corresponding to $\Delta I_{min}$.

and $\gamma_{max} = \gamma_{\perp}$ (when $\theta = \pi/2$), corresponding to $\Delta I_{max}$.

For an oblate spheroid (*m* < 1), the minimum and maximum of γ are:

$\gamma_{min} = \gamma_{\perp} (when\ \theta = \pi/2)$, corresponding to $\Delta I_{min}$.

$\gamma_{max} = \gamma_{\parallel} (when\ \theta = 0)$, corresponding to $\Delta I_{max}$.

This allows us to calculate the electrical shape factor for oblate particles as:

$$\Lambda(\gamma_{\perp}(m), \Delta I_{min}) = \Lambda(\gamma_{\parallel}(m), \Delta I_{max}) \quad \text{(S7)}$$

Thus, the electrical shape factor for prolate particles is given by:

$$\Lambda(\gamma_{\parallel}(m), \Delta I_{min}) = \Lambda(\gamma_{\perp}(m), \Delta I_{max}) \quad \text{(S8)}$$

In both Equations S7 and S8, $\gamma_{\parallel}$ and $\gamma_{\perp}$ depend solely on the *m* value, Thus, by solving this system of equations, we determined both the *Λ* and *m* values for oblate and prolate spheroids.

## References

1 K. Liu, M. Lihter, A. Sarathy, S. Caneva, H. Qiu, D. Deiana, V. Tileli, D. T. L. Alexander, S. Hofmann, D. Dumcenco, A. Kis, J.-P. Leburton and A. Radenovic, *Nano Letters*, **2017**, 17, 4223–4230.

2 S. Liang, Y. Liu, F. Xiang, Z. Yao, W. Zhang and W. Guan, *Advanced Sensor Research*, **2024**, 3, 2300196.

3 Yusko, E. C.; B. R. Bruhn; O. M. Eggenberger; J. Houghtaling; R. C. Rollings; N. C. Walsh; S. Nandivada; M. Pindrus; A. R. Hall; D. Sept; J. Li; D. S. Kalonia; M. Mayer. *Nature Nanotechnology*, **2017**, 12, 360-367.

4 Houghtaling, J.; C. Ying; O. M. Eggenberger; A. Fennouri; S. Nandivada; M. Acharjee; J. Li; A. R. Hall; M. Mayer. *ACS Nano*, **2019**, 13, 5231-5242.



This journal is © The Royal Society of Chemistry 20xx

Please do not adjust margins
